# Enteroscopic hemostasis for bleeding hepatopetal collaterals around a hepaticojejunostomy

**DOI:** 10.1055/a-2955-9801

**Published:** 2026-10-16

**Authors:** Koichi Soga, Satsuki Otani, Ou Takagi, Mayumi Yamaguchi, Masaru Kuwada, Ikuhiro Kobori, Katsutoshi Sugimoto

**Affiliations:** 1 Department of Gastroenterology 26263 Dokkyo Medical University Saitama Medical CenterKoshigayaJapan

Bleeding from ectopic varices following hepatobiliary–pancreatic surgery is rare but potentially life-threatening. 1​ ​2​ An 86-year-old man underwent subtotal stomach-preserving pancreaticoduodenectomy for distal bile duct cancer 2 years previously. He was referred for recurrent hematochezia and transfusion-dependent anemia. Conventional upper and lower gastrointestinal endoscopy at the previous hospital failed to identify the bleeding source. Contrast-enhanced computed tomography revealed local tumor recurrence with complete main portal vein occlusion, cavernous transformation at the hepatic hilum, and collateral vessels around the hepaticojejunostomy site ( Fig. 1 ).

**Fig. 1:**
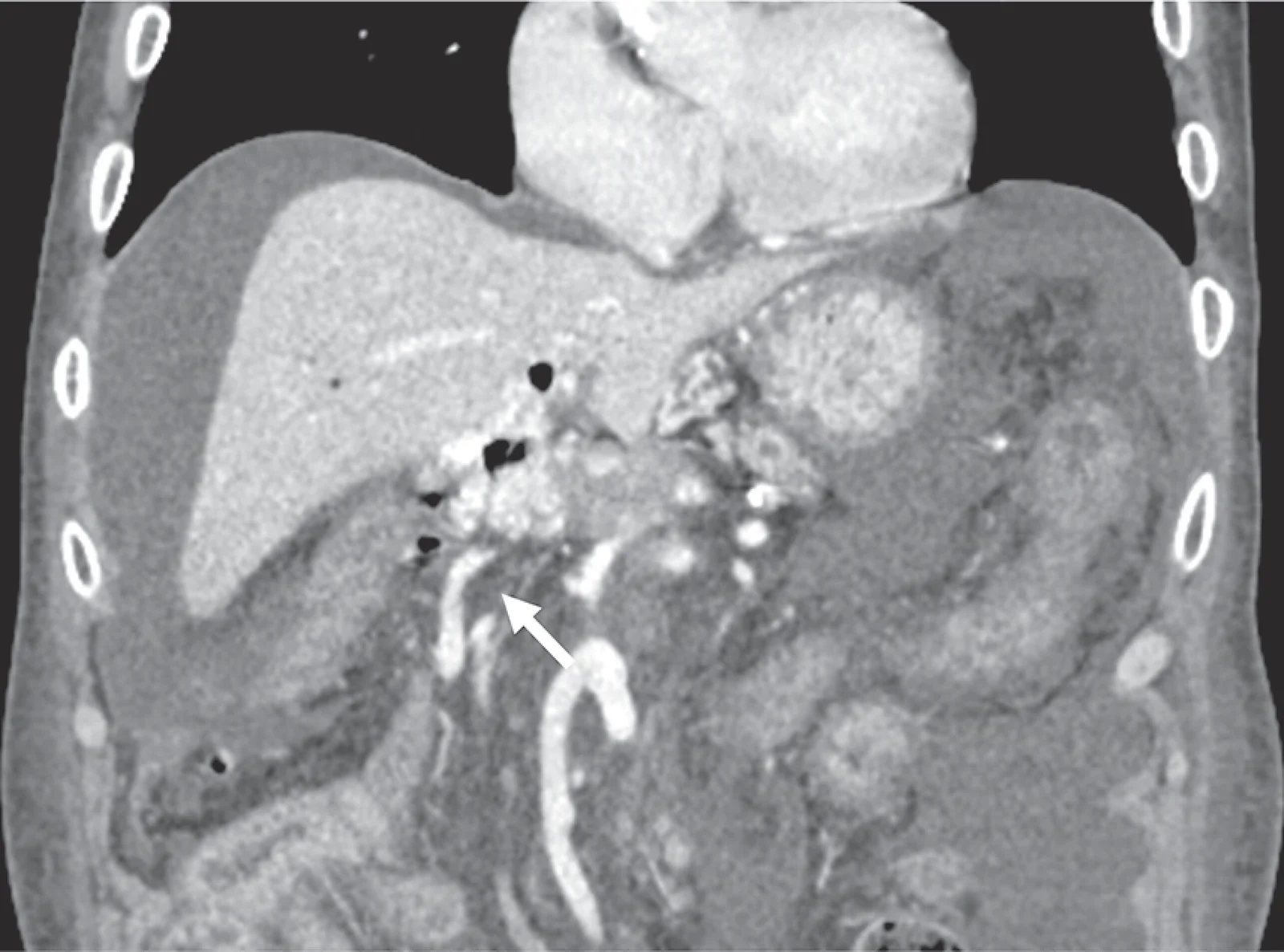
Contrast-enhanced abdominal computed tomographic findings. Contrast-enhanced abdominal computed tomography revealed complete occlusion of the main portal vein owing to tumor recurrence. Cavernous transformation was identified at the hepatic hilum, with hepatopetal collateral pathways developing from the jejunal veins via the region surrounding the hepaticojejunostomy site.

Single-balloon enteroscopy (SIF-H290S; Olympus Medical Systems, Tokyo, Japan) was performed due to suspected bleeding from the biliary-enteric anastomosis. The hepaticojejunostomy was reached, and intermittent bleeding was observed at the anastomotic site. Careful inspection revealed a minute orifice at the anastomotic site with intermittent oozing of blood from the same point. These findings suggested hepatopetal collateral vessel rupture or ectopic varices around the hepaticojejunostomy site secondary to malignant portal vein occlusion. 3​ Endoscopic clipping was performed directly at the bleeding point, followed by the application of a self-assembling peptide hemostatic gel (PuraStat; 3-D Matrix, Tokyo, Japan) which facilitated immediate hemostasis ( Figs. 2 3 and Video 1 ).

**Fig. 2:**
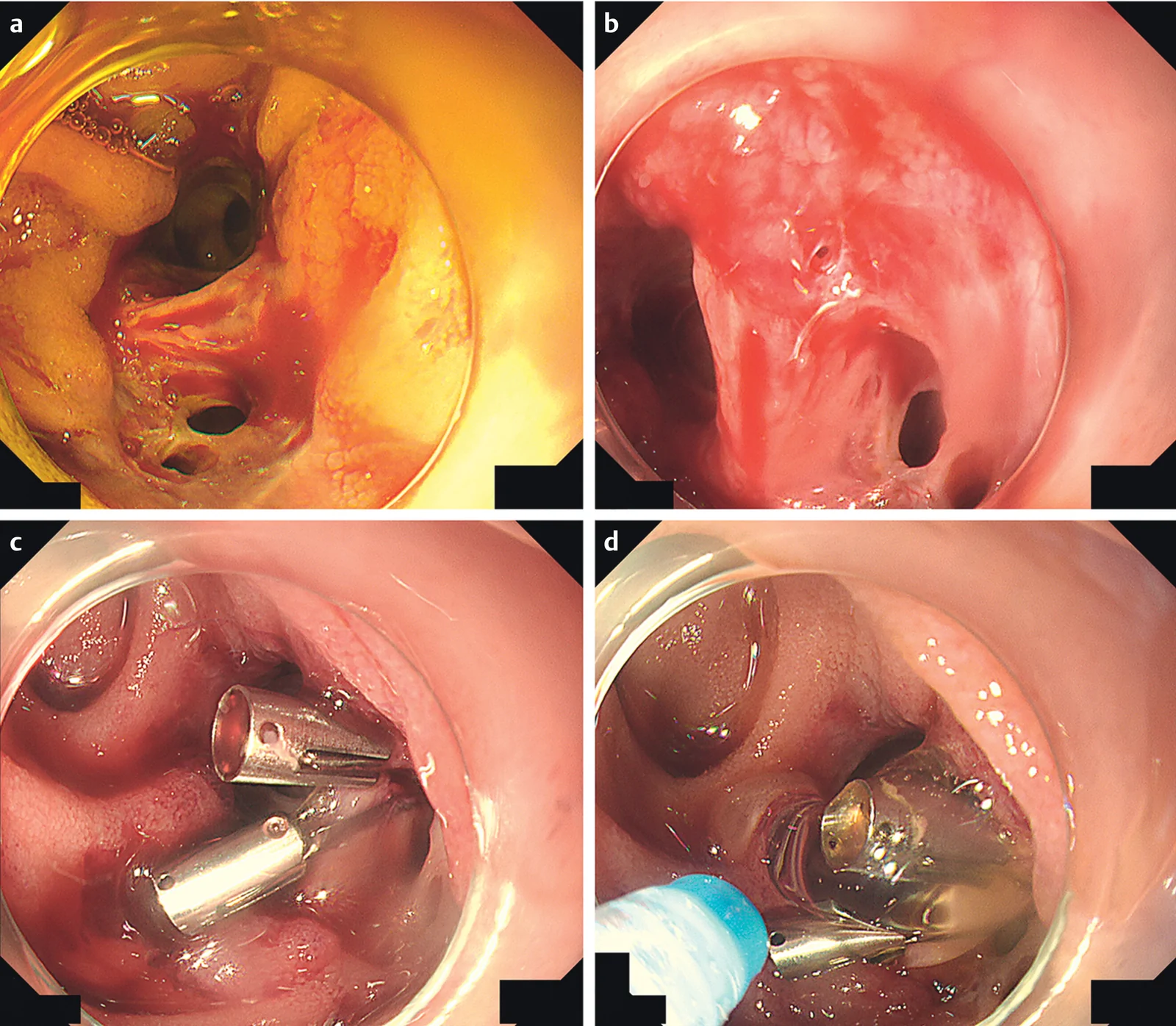
Single-balloon enteroscopic findings of bleeding at the hepaticojejunostomy site and endoscopic hemostasis. ( **a** ) Intermittent bleeding was observed around the hepaticojejunostomy site. ( **b** ) Careful observation revealed a minute orifice at the hepaticojejunostomy site from which intermittent bleeding was observed, suggesting that this site was the source of the hemorrhage. ( **c** ) The bleeding point was closed using hemostatic clips, and immediate hemostasis was achieved. ( **d** ) After clipping, a self-assembling peptide hemostatic gel (PuraStat) was applied to the treated area as an adjunctive hemostatic measure.

**Fig. 3:**
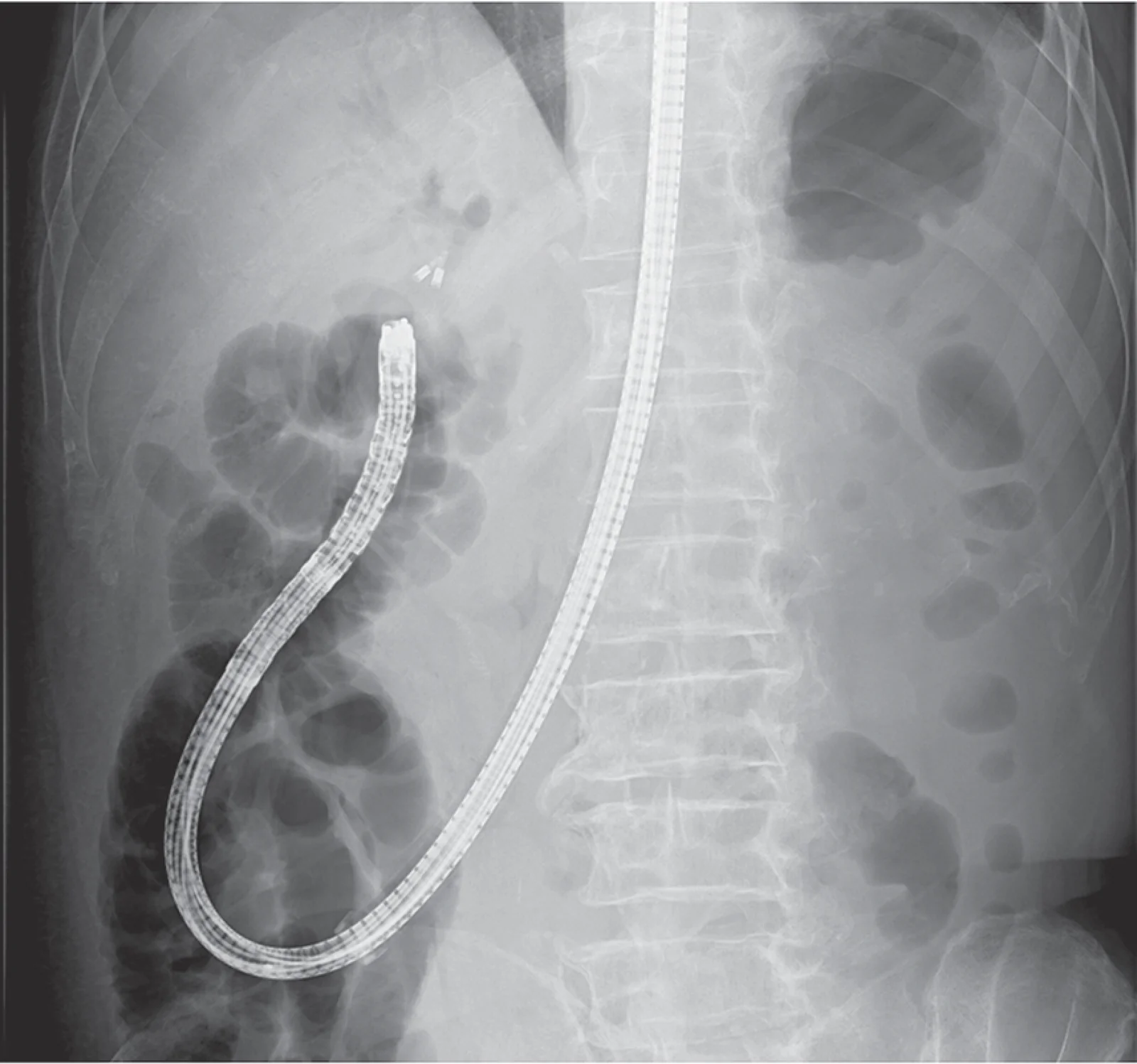
Endoscopic access to the hepaticojejunostomy using a single-balloon enteroscope. A single-balloon enteroscope was advanced into the hepaticojejunostomy site. Endoscopic hemostasis was performed for the bleeding originating from the hepatopetal collateral vessels surrounding the hepaticojejunostomy site.

**Video 1** Single-balloon enteroscopy identified intermittent bleeding from the hepatopetal collateral vessels surrounding the hepaticojejunostomy site, followed by clipping, PuraStat application, portographic confirmation, and portal vein stenting. Video URI:


Subsequent portography demonstrated the complete interruption of flow through the main portal vein. The hepatopetal collateral pathways arising from the jejunal veins coursed through the region around the hepaticojejunostomy toward the hepatic hilum, corresponding to the endoscopic bleeding site. Portal vein stenting was then performed to restore portal inflow and reduce collateral venous pressure. 4​ ​5​ Portal flow was maintained after stenting, and no rebleeding occurred during the 3-month follow-up period until death from the underlying malignancy ( Fig. 4 ).

**Fig. 4:**
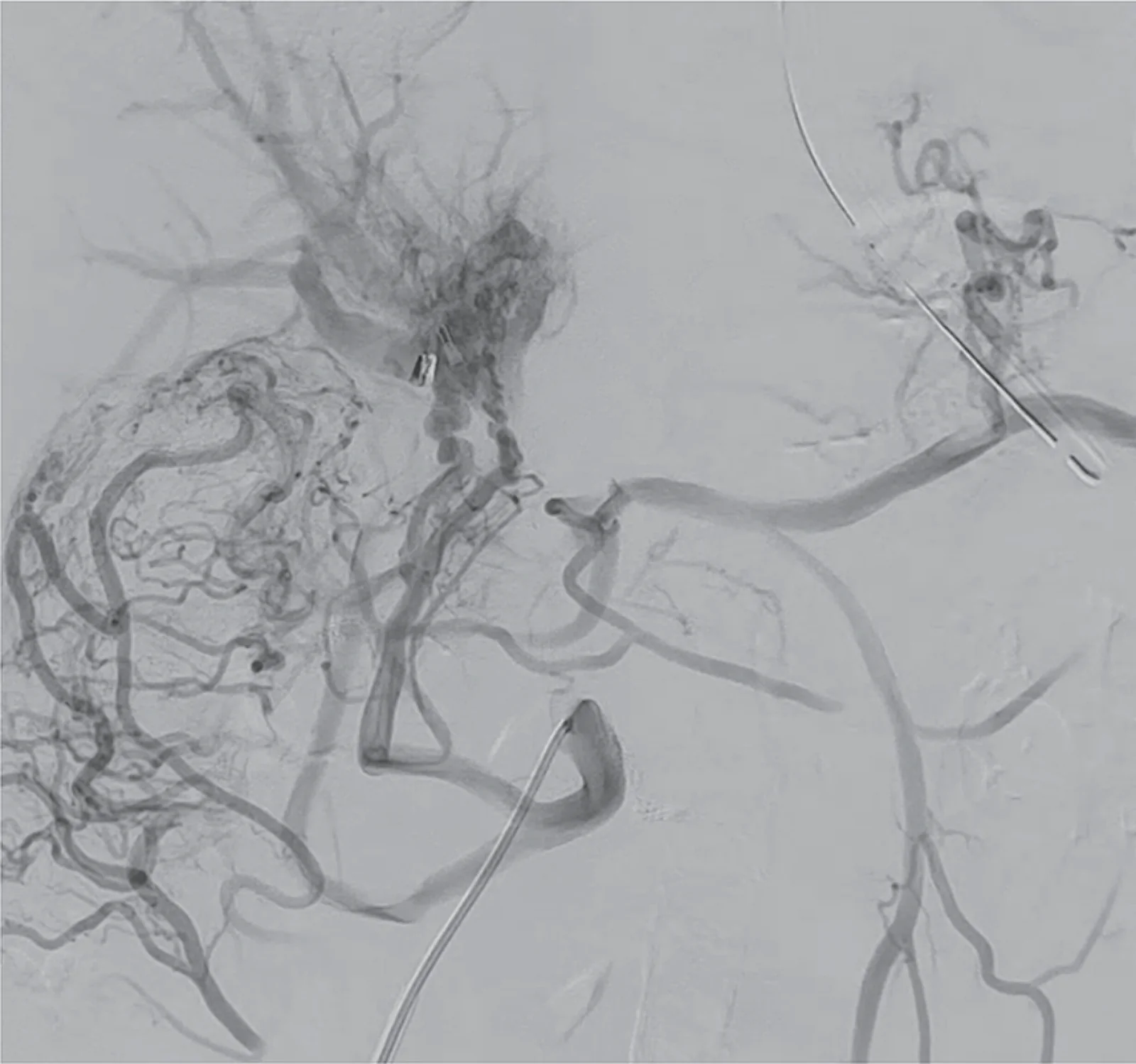
Angiographic findings after endoscopic hemostasis. Post-hemostatic angiography demonstrated the complete interruption of the flow into the main portal vein. In contrast, the hepatopetal collateral pathways arose from the jejunal veins and coursed through the area surrounding the hepaticojejunostomy toward the hepatic hilum, where the cavernous transformation developed.

This case highlights that bleeding from the hepatopetal collateral vessels around a hepaticojejunostomy should be considered in patients with surgically altered anatomy and portal vein obstruction. Single-balloon enteroscopy can directly identify and temporarily control bleeding, thereby bridging the need for definitive hemodynamic therapy.

## Institutional Review Board Approval

Not required for this case report in accordance with institutional policy.

Endoscopy_UCTN_Code_TTT_1AO_2AD

## Declaration of GenAI Use

The authors confirm that no generative artificial intelligence was used in the preparation of this manuscript.
